# Measuring global cerebrovascular pulsatility transmission using 4D flow MRI

**DOI:** 10.1038/s41598-024-63312-4

**Published:** 2024-06-01

**Authors:** Sergio Dempsey, Soroush Safaei, Samantha J. Holdsworth, Gonzalo D. Maso Talou

**Affiliations:** 1 Auckland Bioengineering Institute, University of Auckland, Level 6, 70 Symonds Street, Auckland, 1010 New Zealand; 2 Mātai Medical Research Institute, Tairāwhiti Gisborne, New Zealand; 3 Department of Anatomy and Medical Imaging – Faculty of Medical and Health Sciences & Centre for Brain Research, University of Auckland, Auckland, New Zealand

**Keywords:** Pulsatility, Arterial stiffness, Magnetic resonance imaging, 4D flow, Cerebral blood flow, Blood flow, Cerebrovascular disorders

## Abstract

Pulse wave encephalopathy (PWE) is hypothesised to initiate many forms of dementia, motivating its identification and risk assessment. As candidate pulsatility based biomarkers for PWE, pulsatility index and pulsatility damping have been studied and, currently, do not adequately stratify risk due to variability in pulsatility and spatial bias. Here, we propose a locus-independent pulsatility transmission coefficient computed by spatially tracking pulsatility along vessels to characterise the brain pulse dynamics at a whole-organ level. Our preliminary analyses in a cohort of 20 subjects indicate that this measurement agrees with clinical observations relating blood pulsatility with age, heart rate, and sex, making it a suitable candidate to study the risk of PWE. We identified transmission differences between vascular regions perfused by the basilar and internal carotid arteries attributed to the identified dependence on cerebral blood flow, and some participants presented differences between the internal carotid perfused regions that were not related to flow or pulsatility burden, suggesting underlying mechanical differences. Large populational studies would benefit from retrospective pulsatility transmission analyses, providing a new comprehensive arterial description of the hemodynamic state in the brain. We provide a publicly available implementation of our tools to derive this coefficient, built into pre-existing open-source software.

## Introduction

Abnormal vascular pulsatility is strongly linked to cerebral parenchymal damage and many manifestations of small vessel disease: white matter hyperintensities^1^, brain atrophy^2,3^ and dilated (or enlarged) perivascular spaces^4^. Pulsatility damage, termed pulse wave encephalopathy (PWE) is hypothesised to be rooted in several neurodegenerative diseases, including vascular dementia and Alzheimer's disease^5^. To support this, an increase in pulsatility has been observed with increasing severity of Alzheimer's disease^6–8^ and is associated with increased cognitive impairment^3^. Originated from cardiac contraction, waves of pulsatile pressure travel from the heart to the vascular bed, where the amplitude, speed, and temporal profile strongly depend on the energy-absorbing Windkessel effect of the vessels^9^, the cardiac frequency^10^, the peripheral microvascular resistance^11^, and the cardiac contractility^12^. In the assumed healthy case, this pulsatility transmission is well dampened before reaching the small vessels^13^, although several factors can alter this pulsatility transmission including age^14^, sex^15^, cardiovascular factors (chronotropy, inotropy)^10^, nervous system function^16^, pathological influences such as hypertension^4^ and potentially pathological alterations in peripheral vasculature^11^. These factors increase the variability of pulsatility transmission and can manifest pathological penetration of pulsatility into smaller vessels, where it is hypothesised that encephalopathy occurs with the adjacent parenchyma^5,13^.

Pulsatility is typically measured via the pulsatility index (${p}_{pi}$) proposed by Gosling et al.^17^ based on the measured flow traces (standard), the area traces^18^ or the velocity traces^19^ over the cardiac cycle. Velocity-based ${p}_{pi}$ is the most popular measurement due to its ability to be measured with conventional transcranial doppler (TCD), as well as 2D- and 3D- phase-contrast MRI (2D-PC and 4D flow, respectively). ${p}_{pi}$ represents the relative pulsatility amplitude with respect to the mean flow, giving an idea of the local dynamics, but does not provide a spatial description of how dynamics change throughout the system. Under normal and pathological conditions, ${p}_{pi}$ also presents a wide range throughout the vasculature for healthy and diseased groups^6,14^. This variability is caused by the factors described above and limits the use of ${p}_{pi}$ alone to inform about the PWE risk. Therefore, to better measure the risk of PWE, researchers are also studying the pulsatility damping factor (${p}_{df}$), which is the ratio of ${p}_{pi}$ between the proximal and distal vessels. ${p}_{df}$ is more suitable to assess the risk of PWE as it better represents vascular compliance and the Windkessel effect between two points compared to any single ${p}_{pi}$ measurement alone. This allows for a more informed extrapolation of the pulsatility to reach small vessels not identifiable in the imaging modality of choice.

Damping was first identified in the carotid siphon^20^ and atlas slope^21^, before being studied in the circle of Willis by Zarrinkoob et al.^22^, who analysed damping with age using 2D-PC. Following Zarrinkoob et al., several research works have been produced studying ${p}_{df}$ in the circle of Willis using TCD, 2D-PC and 4D flow^4,7,15,16,18,19,23^. What we highlight based on previous research is that damping has always been measured between two vessel locations, for example, a measurement in the internal carotid artery (ICA) or basilar artery (BA), and a measurement in a distal middle, anterior, or posterior cerebral artery (MCA, ACA, PCA, respectively). Relying on two measurements alone (although still better than ${p}_{pi}$) can easily permit noise contamination if the location happens to be an area of hemodynamic complexity or the collection was not ideal, for example, increased motion during a particular phase of collection or excessive heart rate variability; it can also hide significant changes in the system by being biased by the chosen vasculature. This suggests that ${p}_{df}$ may still not extrapolate well to small vessels to assess the risk of PWE. Inter-subject comparison is also difficult due to challenges in identifying the same or equivalent loci for the measurements due to anatomical differences and technological limitations (e.g., reliably locating TCD planes of acquisition in the same place of the anatomy).

These limitations are understandable in TCD and 2D-PC: TCD has a limited ability to detect distal arteries, and 2D-PC protocol limits the ability to sample more than a handful of points. However, 4D flow does not suffer these limitations; it provides simultaneous velocity measurement in all identifiable cerebrovasculature. So far, in terms of ${p}_{df}$, 4D flow has been used only in the same way as 2D-PC and TCD by selecting only two points^7,18^, which does not take advantage of the rich information collected. It was always the anticipation of Zarrinkoob et al. that 4D flow would be leveraged for a more robust damping measurement, and here we achieve this goal.

This work presents a novel measurement of pulsatility transmission, ${p}_{tc}$, that integrates whole-organ 4D flow MR data leading to a robust characterisation of the subject-specific arterial pulsatility state. This new index is measured by characterising how ${p}_{pi}$ changes with increasing distance into the peripheral vascular tree using hundreds of cross-sectional ${p}_{pi}$ measurements instead of one or two in ${p}_{pi}$ and ${p}_{df}$, providing a more confident pulsatility-based index. We present analysis of ${p}_{tc}$ sources of error, and how ${p}_{tc}$ measurement trends with age, heart rate, sex, vascular symmetries, and cerebral blood flow (CBF) compared to already established ${p}_{pi}$ and ${p}_{df}$, and discuss applications in health and modelling.

## Materials and methods

### Subjects and scanning protocol

To develop and assess this index, we retrospectively analysed two cohorts with 4D flow data. Ethical approval was obtained from the New Zealand Health and Disability Ethics Committee (HDEC9430) and the University of Auckland Ethics Committee (AHREC1006); the study was performed in accordance with the guidelines of the 1964 Declaration of Helsinki and its later amendments. All participants provided written informed consent. 4D flow data from 20 healthy participants (age 48 ± 18 years; 10 female, 10 male) were available. The inclusion criteria were those without a history of brain trauma, hypertension, cognitive impairment, or cardiovascular disease.

The images were acquired at Mātai Medical Research Institute using a 3T MRI scanner (GE SIGNA Premier; General Electric Healthcare, Milwaukee, WI, USA) with an AIR™ 48 channel head coil. 4D flow data were acquired using a k-adaptive-t auto calibrating reconstruction for cartesian sampling (kat-ARC)^24^ 3D phase-contrast sequence with imaging parameters shown in Table 1. Several works show that at resolutions ≤ 1mm isotropic, the flow closely approximates 2D-PC, the clinical gold standard for intracranial flow analysis^25–29^. The pulsatility measurements on which this work is based have also been shown to be insensitive to resolution and partial volume effects^28^.

**Table 1:** 4D flow scan parameters for the cohort studied.

Scan parameters	Value
Scanned resolution	1 × 1 × 1 mm
Reconstructed resolution from zero padding k-space	0.5 × 0.5 × 0.5 mm
# of cardiac phases	20
Cardiac synchronisation	Retrospective Pulse Oximeter
Flip angle	8°
Acquisition	3D
Encoding velocity	80 cm/s
Echo time	Approximately 3 ms
Repetition time	Approximately 5.4 ms
Signal averages	4
Acquisition time	Approximately 10 min
Inferior-superior FOV	Carotid canal to ~ 4cm above circle of Willis

### 4D flow processing

**Figure 1:**
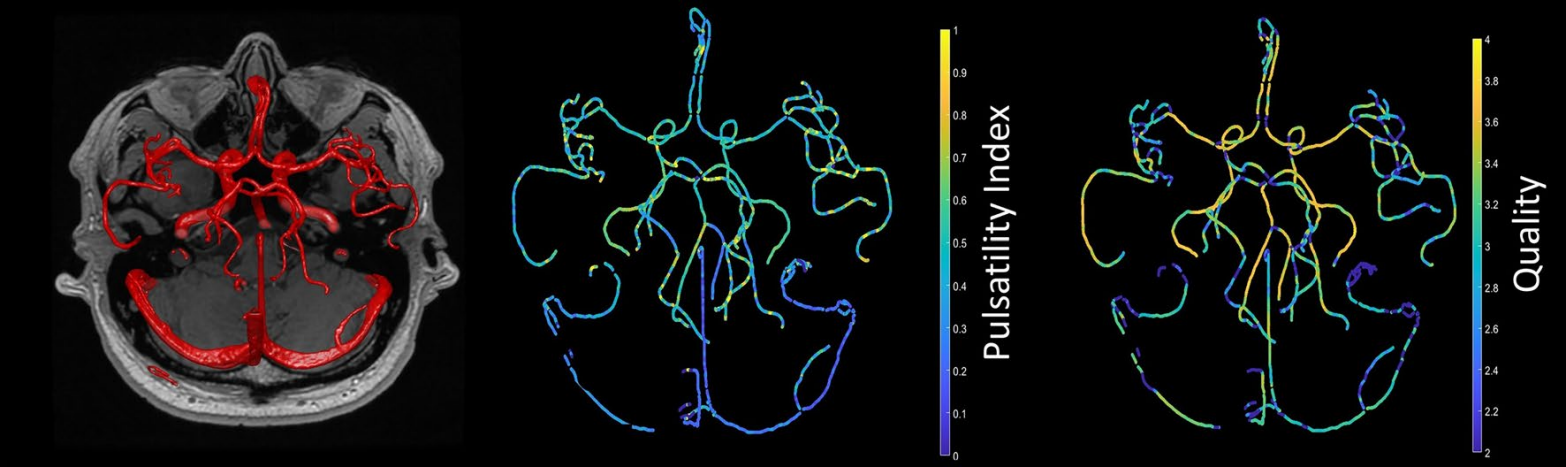
Example iso-surface of QVT segmentation from 4D flow (left) and visualisation of ${p}_{pi}$ (centre) and processing quality computed via Eq. 4 (right).

The 4D flow data was processed using a modified Quantitative Velocity Tool (QVT) (https://github.com/uwmri/QVT), a semi-automated software that computes cross-sectional vessel flows from 4D flow data^26,30^. QVT performs background correction and automatically segments the vasculature, computes the tangent derivatives of the vessel centrelines, interpolates the cross-sectional vessel planes, performs in-plane segmentation, and finally computes flow and other hemodynamic indices from plane-normal vessel velocities. The output of standard QVT analysis, including segmentation and measurement of ${p}_{pi}$, is shown in Fig. 1. QVT also offers a user-friendly interactive GUI to probe 4D flow-based measurements. We have modified the software, hereafter called QVT+, to include the acceptance of DICOM formatted 4D flow data, to compute the proposed ${p}_{tc}$, and to include other functionalities described in later sections. QVT+ software is developed in Matlab v2023a (MathWorks, Natick, MA, USA) and is available at https://github.com/ABI-Animus-Laboratory/QVTplus.

### Measuring pulsatility transmission

To present how the ${p}_{tc}$ index is measured, we define the set of all vessel centreline points as $\mathcal{V}$ and the blood flow field $q:\mathcal{V}\times [1,S]\to {\mathbb{R}}$, where $S$ is the number of measurements performed for $q$ – e.g., in a 4D flow MRI, $\text{S}$ is the number of cardiac phases during the cardiac period $T$. Gosling's ${p}_{pi}$ with this formalism is defined as

1 $$\begin{array}{c}{p}_{pi}\left(\mathbf{x}\right)=\frac{{q}_{max}\left(\mathbf{x}\right)-{q}_{min}\left(\mathbf{x}\right)}{{q}_{mean}\left(\mathbf{x}\right)} \end{array}$$

where $q\left(\mathbf{x},t\right)$ is the flow at the spatial location $\mathbf{x}$ and at time $t\in \left[1,S\right]\subseteq {\mathbb{Z}}$, ${q}_{max}\left(\mathbf{x}\right)$, ${q}_{min}\left(\mathbf{x}\right)$, and ${q}_{mean}\left(\mathbf{x}\right)$ are respectively the minimum, maximum and mean flow at the spatial location $\mathbf{x}$ during the cardiac cycle. Pulsatility damping, ${\text{p}}_{\text{df}}$, is defined as

2 $$\begin{array}{c}{p}_{df}({\mathbf{x}}_{d},{\mathbf{x}}_{p})=\frac{{p}_{pi}\left({\mathbf{x}}_{d}\right)}{{p}_{pi}\left({\mathbf{x}}_{p}\right)}\end{array}$$

where ${\mathbf{x}}_{\text{p}}$ and ${\mathbf{x}}_{\text{d}}$ are a proximal and a distal spatial location along the direction of blood perfusion.

For our proposed index, we first evaluate ${p}_{pi}(\mathbf{x})$, $\forall \mathbf{x}\in \mathcal{V}$, i.e., at all centreline positions of the observable vascular tree. Then, based on a set of ${p}_{pi}$ pulsatility measurements, we define the pulsatility transmission function ${p}_{tf}(d)$ as a continuous function that estimates how the pulsatility changes throughout the vascular tree based on distance $(d)$. The ${p}_{tf}$ function is approximated as a linear function

3 $$\begin{array}{c}{p}_{tf}\left(\mathbf{x}\right)={p}_{tc}d\left(\mathbf{x},{\mathbf{x}}^{r}\right)+\beta \end{array}$$

where $d\left(\mathbf{x},{\mathbf{x}}^{r}\right)$ is the Euclidean distance from an initialisation root vessel ${\mathbf{x}}^{r}$ $(d=0)$ to a downstream distal point $\mathbf{x}$ over the connecting centreline. $\beta$ is an offset and ${p}_{tc}$ is the slope that we define as our transmission coefficient measurement. Notice that ${p}_{tf}$ depends on ${\mathbf{x}}^{r}$, but we drop the dependency for the sake of readability. The linear assumption in Eq. 3 is based on the work of Padmos et al.^31^ (see Fig. 3D in that work), which presents evidence of ${p}_{pi}$ decreasing linearly with distance from the heart in a computational model of the cerebrovasculature.

To define the function $d\left(\mathbf{x},{\mathbf{x}}^{r}\right)$, we must determine the centrelines connecting ${\mathbf{x}}^{r}$ to all $\mathbf{x}$ downstream. However, QVT only provides an unlinked representation of the vessels, so a breadth-first connection algorithm was developed to resolve the vessel connectivity at all bifurcation points (see details in the Supplementary Note 1). Briefly, starting with the root vessel ${\mathbf{x}}^{r}$, its terminal is connected to nearby vessel inlets within an 8-voxel distance. As shown in Sect. 3.1 the empirical value of 8 pixels yields good connectivity while minimising the occurrence of invalid connections. The terminals of these newly connected vessels then initiate a new inlet search round until no new vessels are found. At times, the vessel network has vessel crossovers/kissing issues, previously reported during the analysis of 4D flow-based vascular trees^32^. It is difficult to confidently determine vascular connectivity when this occurs, so connections that yield the more regular solution are chosen, i.e., when centreline tangents at the connected inlet and outlet maximise their inner product (maximising alignment). For the vessels now connected to ${\mathbf{x}}^{r}$, $d\left(\mathbf{x},{\mathbf{x}}^{r}\right)$ is calculated by adding the Euclidean distance between subsequent centreline points for vessels connecting ${\mathbf{x}}^{r}$ to each $\mathbf{x}$ in the set.

We acknowledge that a common occurrence with full automation of 4D flow processing is that some measurements may be corrupted by incorrect segmentation and noisy flow and, as such, some ${p}_{pi}\left(\mathbf{x}\right)$ values, may not have acceptable quality for fitting ${p}_{tf}\left(\mathbf{x}\right)$. It is possible to correct poor automatic vessel segmentations with manual tracing, but with thousands of cross-sections to check for each subject, this is infeasible. Therefore, we have also included a quality function processed in QVT + to inform the probability that a calculated ${p}_{pi}$ is acceptable for analysis. This quality $Q$ is the sum of four quality metrics based on vessel circularity (reciprocal of eccentricity), local mass, area, and flow trace conservation, defined as

4 $$\begin{array}{c}Q\left(\mathbf{x}\right)={\mu }_{circ}+\left(1-\frac{{\sigma }_{{q}_{mean}}}{{\mu }_{{q}_{mean}}}\right)+\left(1-\frac{{\sigma }_{area}}{{\mu }_{area}}\right)+\left(1-\frac{\Delta q}{{\mu }_{{q}_{mean}}}\right).\end{array}$$

The first term, circularity, is defined as the ratio of minimum to maximum radius of the segmented cross-section. The second and third terms are deviations of mean flow and area, respectively. The fourth term measures the flow trace conservation with $\Delta q$ defined as

5 $$\begin{array}{c}\Delta q\left({\mathbf{x}}_{i}\right)=\frac{1}{S}\sum_{t=1}^{S}\left(\underset{-2\le j\le 2}{\text{max}}\left(q\left({\mathbf{x}}_{i+j},t\right)\right)-\underset{-2\le j\le 2}{\text{min}}\left(q\left({\mathbf{x}}_{i+j},t\right)\right)\right)\end{array}$$

and effectively measures the overall waveform agreement. ${\mu }_{(\cdot )}$ and ${\sigma }_{(\cdot )}$ are defined as the mean and standard deviation of the physical quantity $(\cdot )$ on a stencil of 5 points centred on the point of interest, i.e., a neighbourhood of 2 points in each direction of the centreline. This stencil length is based on the work of Roberts et al.^30^, who found that 5 points were optimal to remove measurement noise while still capturing local flow metre measurements in a flow phantom. Therefore, within this window, we expect that the 4D flow metrics should be relatively consistent, indicating a high-quality collection.

Based on the quality metric, ${p}_{tc}$ and $\beta$ are optimised to minimise the quality-weighted square error between ${p}_{tf}$ and ${p}_{pi}$ for all $\mathbf{x}$ in a given subvascular tree rooted at ${\mathbf{x}}^{r}$, formally,

6 $$\begin{array}{c}\left({p}_{tc},\beta \right)=\underset{\left({\widehat{p}}_{\mathit{tc}},\widehat{\beta }\right)\in {\mathbb{R}}^{2}}{\text{arg min}}\sum_{\mathbf{x}\in {\mathcal{V}}_{{\mathbf{x}}^{r}}}w\left({\mathbf{x}}\right){\left({\widehat{p}}_{tc}d\left({\mathbf{x}}^{\text{r}},\mathbf{x}\right)+\widehat{\beta }-{p}_{pi}\left(\mathbf{x}\right)\right)}^{2}\end{array}$$

where ${\mathcal{V}}_{{\mathbf{x}}^{r}}$ is the set of all downstream $\mathbf{x}\in \mathcal{V}$ to ${\mathbf{x}}^{r}$ with $Q\ge 2.5$, and

7 $$\begin{array}{c}w\left(\mathbf{x}\right)=\frac{Q\left(\mathbf{x}\right)-2.5}{{Q}_{max}-2.5}\end{array}$$

is the weight. We first discard any measurement from ${\mathcal{V}}_{{\mathbf{x}}^{r}}$ where $Q<2.5$, which were almost always unacceptable data (see Sect. 3.2 and the Supplementary Figure S3 for analysis of the threshold quality value). The remaining ${p}_{pi}\left(\mathbf{x}\right)$ are then assigned weights based on $Q$, where the weight value is linearly distributed from 0 to 1 for $Q\in 2.5,{Q}_{max}$ where ${Q}_{max}=4$. The weights ensure confident values of ${p}_{pi}$ better influence the fit.

For our analysis, ${p}_{tc}$ is measured for the three main subvascular trees performing cerebrovascular perfusion, specifically, ${\mathbf{x}}^{r}$ being the root of the left and right ICAs, and the BA ignoring connections formed by the communicating arteries. To measure global organ-level transmission, the three network ${p}_{tc}$ values were averaged. Details of the algorithm implementation and user initialisation are present in the Supplementary Note 1, Supplementary Figure S1, and in the GitHub repository.

### Measuring ${{\varvec{p}}}_{{\varvec{p}}{\varvec{i}}}$ and ${{\varvec{p}}}_{{\varvec{d}}{\varvec{f}}}$ indices

We now present the methodology for measuring previously developed ${p}_{pi}$ and ${p}_{df}$ that we will use to compare with ${p}_{tc}$ for physiological trends. ${p}_{pi}$ was measured at the middle of the C3 segment for the ICAs, and the same height for the BA root. A global ${p}_{pi}$ was calculated as the mean of all three roots. These root pulsatilities were also considered as the ${p}_{pi}({\mathbf{x}}_{p})$ to compute ${p}_{df}$. For the ICAs, ${p}_{pi}({\mathbf{x}}_{d})$ was the mean ${p}_{pi}$ of the start of the M1 and A1 segments. For the BA, the left and right P1 segments at the initial posterior bend were averaged for ${p}_{pi}({\mathbf{x}}_{d})$ (see Fig. 3 in Roberts et al.^30^ for a visualisation of sample locations in the vascular tree). For all selected pulsatility locations, a mean ${p}_{pi}$ with a 5-point window was used, which is recommended for QVT flow quality^30^.

Concerning anatomical variation, for example, in case of a missing A1 segment because one ICA bifurcates both ACAs; only a single point was used for ${p}_{df}$. If a root ${p}_{pi}$ was missing, that root was omitted from the global average of ${p}_{pi}$, and ${p}_{df}$. This anatomical variation does not limit ${p}_{tc}$, which is locus independent.

## Results

### Analysing connectivity

We first confirmed that any errors in the automatic vessel connectivity were within acceptable ranges. This required quantifying any ${p}_{tc}$ error associated with incorrect distances $d\left(\mathbf{x},{\mathbf{x}}^{r}\right)$. Distance errors can arise from assuming a straight line-based connection between vessels, ignoring potential curvature or from incorrectly connected kissing and crossing vessel continuities. The incorrect connections lead to distance shifts of downstream vessels based on the difference in distance along the true and assigned vessel connectivity to $\mathbf{x}$**.** To evaluate this error, we generated synthetic ${p}_{pi}$ data with random shift errors between vessel continuations (for more details on the simulation, see Supplementary Note 2 and Figure S2). We simulated 1000 cases and fit ${p}_{tf}$ using Eq. 6 with $w(\mathbf{x})=1$. Compared to the ground truth ${p}_{tc}$ of − 1 in our simulation, distance errors resulted in a ${p}_{tc}$ of − 0.99998 ± 0.027. Given the symmetric error distribution, we conclude that compounded distance shifts do not bias the results. The symmetry in the error is expected in practice since there is no preference to distance elongations or contractions as vessel connections are randomly ordered.

The default connectivity algorithm parameters worked for most cases to connect the vessel networks completely, which were all manually checked afterwards. Sometimes, the gaps between the vessels were larger than the default search distance assigned. This was common in the BA when connecting to the PCAs since the superior cerebellar arteries can cause large gaps in the QVT vessel junctions. Once the search distance was increased to connect the BA to PCAs, in all cases, the rest of the vasculature was fully connected. We were reluctant to increase the default search distance as the larger search may create spurious connections with distant vessels. The entire process to initialise the algorithm and check the connectivity interactively in the QVT+ module took ~ 1 min per case.

### Analysing quality

We found a positive impact of quality thresholding and weighting. Overall, the use of $Q$ as a threshold removed a minimal amount of data while removing a large amount of noise at the vessel junction ends. It also highlighted the similar processing quality of ICA and BA data. The impact of $w\left(\mathbf{x}\right)$ on fitting once the low-quality data were thresholded was minimal, showing a low measurement dependency on external processing factors.

To assess the quality impact, we analysed the included and excluded ${p}_{pi}$ samples in terms of remaining occupancy, quality $Q$, pulsatility ${p}_{pi}$, location of low quality, root differences, and impact on ${p}_{tc}$ fit. Globally, the quality threshold preserved most of the data extracted from 4D flow (87 ± 4.3% of the samples), presenting a quality of $Q$=3.3 ± 0.27 across 1597 ± 421 cross-sections (a minimum of 910 and a maximum of 2493) included per participant. In terms of pulsatility, after excluding low-quality data, a more consistent pulsatility (${p}_{pi}$=0.55 ± 0.12 with a within subject variation of 0.02) remained, while excluded data presented a wider spread of measurements (${p}_{pi}$= 0.78 ± 3.7 with a within subject variation of 3.2) due to incorrect segmentation and noisy velocity effects. The location of this low-quality data was explored, and it was observed that 52.8% of the $Q$ < 2.5 data was in the first and last 15% of the vessels and was otherwise evenly identified within the remaining 70%. This shows that the discarded measurements were located mainly at vessel bifurcations, which would be expected since the adjacent vessels are close and likely to affect centreline vessel segmentation. Low quality within the vessel was typically caused by poor segmentation or low velocity SNR, impacting local waveform tightness.

The roots of ICA versus BA showed minor differences; 85 ± 4.3% ICA data versus 91 ± 5.2% BA data remained after thresholding with a remaining $Q$ of 3.3 ± 0.27 and 3.27 ± 0.27, respectively. A greater number of cross-sections was observed for the ICAs, with an average of 555 ± 149 versus 487 ± 160 for the BA. This is expected, as the ICA vascular trees are longer than the BA tree and with two major bifurcations (MCA and ACA) contributing to the data. In support of this, the maximum distances were 180 ± 44mm versus 122 ± 33mm for ICAs and BA, respectively.

To explore the impact on ${p}_{tc}$ fit, we calculated ${p}_{tc}$ without $w(\mathbf{x})$ in Eq. 6 and with or without excluding $Q$ < 2.5. Without $w(\mathbf{x})$ but excluding $Q$ < 2.5, the ${p}_{tc}$ difference was 2.5 ± 5% relative to the calculated root slope range (− 1.6 to 1.9 $({p}_{pi}/m)$) which is minor for the observations in the next section. Without $w(\mathbf{x})$ and including $Q$ < 2.5, the slope differences were − 1.1 ± 63.5% relative to the calculated root slope range again. This massive variability is explained by the clear influence of noisy ${p}_{pi}$ data within $Q$ < 2.5 on the fit, which highlights why exclusion was necessary. $w(\mathbf{x})$ was used in the remaining sections as we believe emphasising high quality is still appropriate.

### Analysing indices qualitatively

**Figure 2:**
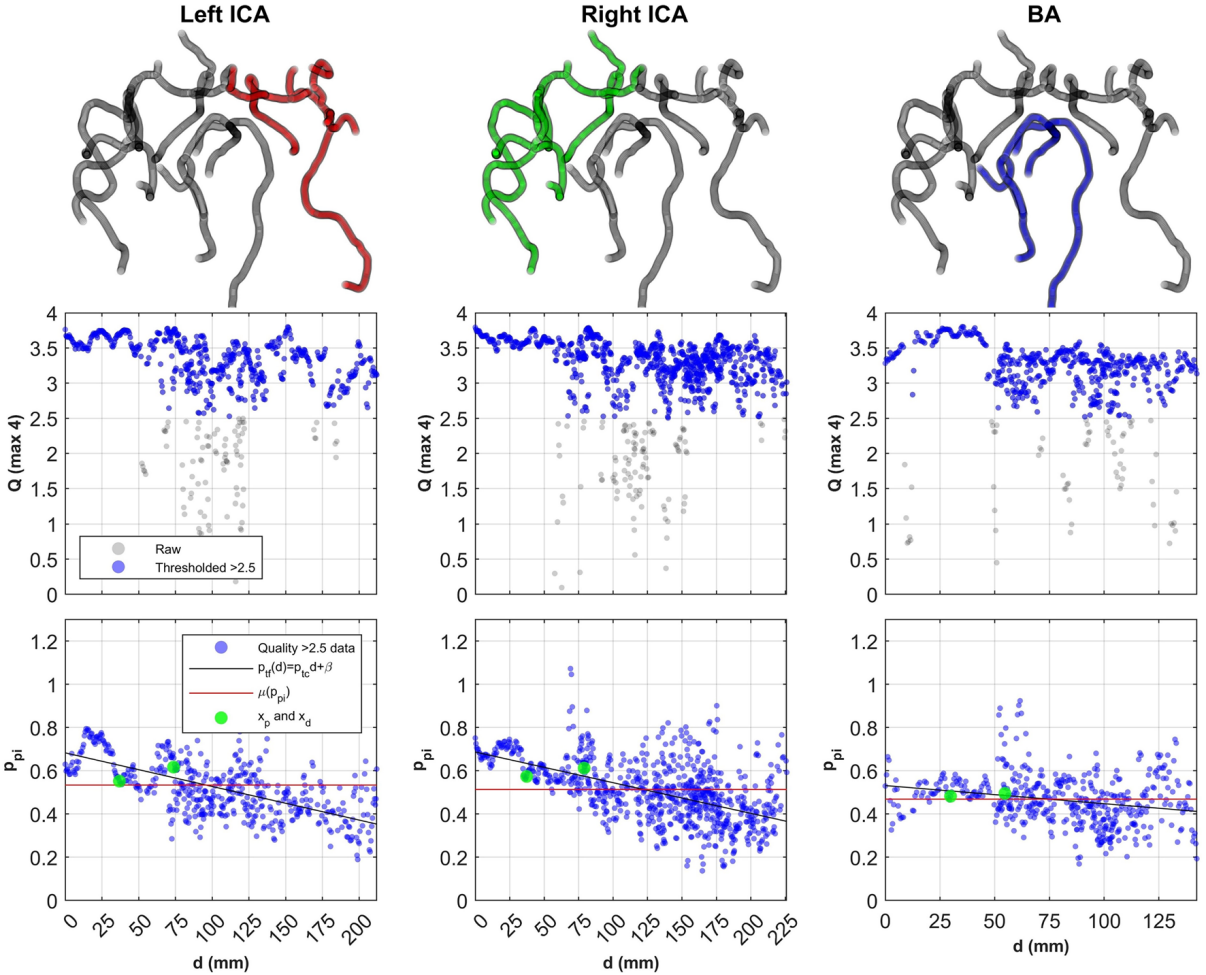
Visualisation of the individual root network results for a nominal participant. The network for each root is visualised from an inferior position along the inferior-superior axis (top row). Root network quality scores, $Q$, (middle row) and pulsatility, ${p}_{pi}$ (bottom row) at different distances, $d\left(\mathbf{x},{\mathbf{x}}^{r}\right)$ for left and right ICAs and BA are then plotted. Overlaid measurement values for ${p}_{pi}$, ${p}_{df}$ , and ${p}_{tc}$ are presented in the bottom row for comparison. As ${p}_{tc}$ integrates all high-quality ${p}_{pi}$ samples, it provides a more comprehensive description of the pulsatility for each subvascular tree. Abbreviations: basilar artery (BA), distance (d), internal carotid artery (ICA), quality (Q).

The linear approximation for ${p}_{tf}$ seems qualitatively appropriate, although a weighted R-squared value of 0.05 suggests otherwise. We attribute this to the spatial variability of pulsatility present in the distal regions (see Fig. 2). As discussed in the introduction, individual samples of ${p}_{pi}$ and ${p}_{df}$ were prone to spatial bias misrepresenting the organ level trend of pulsatility (see Fig. 2) which predicts a poorly generalised damping trend compared to our ${p}_{tc}$.

### Analysing indices against covariates

${p}_{pi}$, ${p}_{df}$ and ${p}_{tc}$ are now compared against potential covariates that may influence the measurement, such as age, heart rate, sex, and other exploratory measures, magnitude of flow, velocity, and quality. At this stage, since the sample size is small, the analysis will remain on a per covariate basis, i.e., no generalised linear models will be fit with all covariates since a cohort of hundreds should be used. We first interpret individual root (${\mathbf{x}}^{r}$) trends as the ICAs may behave differently than the BA, we then consider trends of all roots combined, and then a global, averaged root index.

#### Individual root trends of age, heart rate, and sex

First, concerning anatomical variation, of the 20 participants, two had missing right A1 segments, and a third participant's left ICA C3 segment could not be identified confidently. This resulted in one participant's left ICA ${p}_{pi}$ and ${p}_{df}$ not being calculated for root-specific differences.

**Figure 3:**
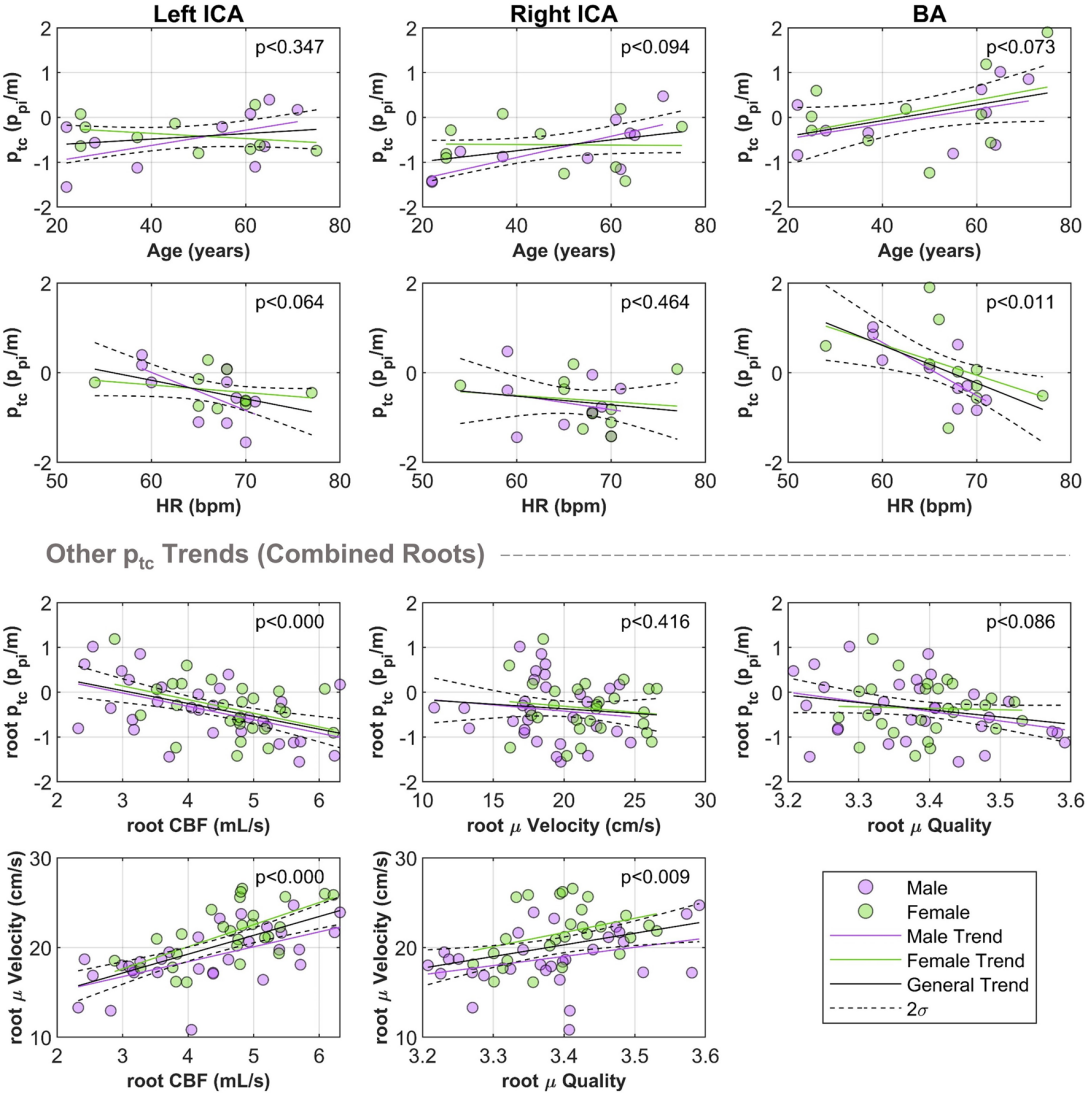
Individual root trends of ${p}_{tc}$ with respect to age (row one), heart rate (row two) for the left ICA (left), right ICA (centre), and BA (right). All roots are then merged for other comparisons against mean CBF, velocity, and quality (bottom two rows). Abbreviations: basilar artery (BA), cerebral blood flow (CBF), heart rate (HR), internal carotid artery (ICA).

For ${p}_{tc}$, we identified that the BA appears to have a larger transmission than ICAs, but trends similar to the ICAs with age, although not significantly (see Fig. 3). The BA transmission significantly dependent on heart rate. Concerning sex, significant trends were observed only for males in the right ICA for age, and BA for heart rate. Interestingly, each root trend showed that females had a larger ${p}_{tc}$ in mid-life. Combining all the root data for trend analysis identified that both age and heart rate were significantly correlated with ${p}_{tc}$, influenced by the significant trend observed in males.

Occasionally, there were ${p}_{tc}$ differences in the left and right ICAs. To explore their cause, the dependency of ${p}_{tc}$ differences with respect to percent left–right differences in CBF and ${p}_{pi}$ root burden was examined (see Supplementary Figure S4). A very minor positive correlation was observed for both.

Concerning local pulsatility, ${p}_{pi}$ had a significant positive correlation with age in the right ICA and BA, though female data had a significant positive correlation for all roots. The combination of all roots identified a significant positive trend again, influenced significantly by females. No significant heart rate trends were observed. See Supplementary Figure S5 for plots of ${p}_{pi}$. We lastly note that females had lower root ICA ${p}_{pi}$ when young, which increased significantly faster with age than in males.

Concerning root damping, ${p}_{df}$ trended positively with age for the ICAs and the BA, but nothing was significant (see Supplementary Figure S6 for plots). The right BA ${p}_{df}$ had a significant negative correlation with heart rate and combining all roots only presented a significant negative heart rate correlation in males.

#### Individual root trends of flow, velocity, and quality

With the presentation of a new index and considering the scale of integration, we examine other covariates that have not been explored in literature, particularly, CBF, velocity, and our own predicted vessel quality. CBF of the network was defined as the mean flow for each initialised root vessel location. Because the vascular depths are variable per participant, to compare velocity and quality between groups, we averaged velocity and quality over the first 100 mm for each root which was fully covered for most participants. This covered the depth for both proximal and distal ${p}_{df}$ locations.

We observed a significant negative correlation of ${p}_{tc}$, with CBF when combining all roots which was significant for both sexes as well. While a significant positive correlation was also observed with CBF and velocity, no correlation of ${p}_{tc}$ with velocity was observed. We observed no significant correlation of ${p}_{tc}$ with quality, although at lower mean quality, an increase in the dispersion of ${p}_{tc}$ was visible (see Fig. 3). There was also a significant positive correlation of velocity with quality. Females had a significantly higher mean velocity of 21.6 ± 3.1cm/s than males with 18.8 ± 3.2cm/s, but no significant difference in root CBF (female CBF of 4.6 ± 0.8mL/s, male CBF of 4.2 ± 1.2mL/s). The quality of both sexes was identical with a mean of 3.4, although females had less variability in quality.

${p}_{pi}$ showed no correlation with CBF, velocity, individually or with combined roots (though minor root dependant sex trends were significant). There was a significant correlation of ${p}_{pi}$, with quality in the ICAs and combined networks, attributed to females.

${p}_{df}$ was significantly negatively correlated with CBF when all roots were combined which remained significant in male-only combined data; the same trends were observed for quality. No correlation of ${p}_{df}$ with velocity was observed. A table summarising the p-values for each trend is available in Table 2.

**Table 2:** *p* values of all slopes for each index regressed against age, heart rate, sex, CBF, and velocity for each vascular root and combined. Significant values at a threshold of *p* < 0.05 are bolded. *p* values first show the general trend, and then bracketed (male|female) trend. Abbreviations: basilar artery (BA), cerebral blood flow (CBF), heart rate (HR), left internal carotid artery (LICA), right internal carotid artery (RICA).

	*p* value of trend			
	LICA	RICA	BA	LICA + RICA + BA
$${p}_{pi}$$				
Age	0.09 (0.67|**0.04**)	**0.01** (0.96|**0.00**)	**0.04** (**0.02**|**0.00**)	**0.00** (0.15|**0.00**)
HR	0.69 (0.22|0.61)	0.63 (0.82|0.69)	0.76 (0.20|0.22)	0.70 (0.10|0.61)
CBF	0.28 (**0.02**|0.19)	0.38 (0.68|**0.05**)	0.62 (0.78|0.15)	0.98 (0.09|**0.04**)
Velocity	0.86 (0.74|0.41)	0.10 (0.38|0.07)	0.11 (0.47|0.12)	0.88 (0.82|0.60)
Quality	**0.03** (0.15|0.14)	**0.03** (0.80|**0.01**)	0.82 (0.67|0.31)	**0.01** (0.98|**0.00**)
$${p}_{df}$$				
Age	0.36 (0.66|0.24)	0.29 (0.95|0.46)	0.17 (0.18|0.74)	0.08 (0.08|0.81)
HR	0.34 (0.13|0.59)	0.59 (0.34|0.14)	**0.02** (**0.04**|0.10)	0.13 (**0.00**|0.33)
CBF	0.36 (0.37|0.74)	0.14 (0.23|0.49)	0.10 (0.10|0.79)	**0.02** (**0.05**|0.49)
Velocity	0.75 (0.74|0.66)	0.76 (0.50|**0.04**)	0.10 (0.09|0.93)	0.65 (0.48|0.66)
Quality	0.28 (0.70|**0.05**)	0.10 (**0.05**|0.83)	0.56 (0.39|0.16)	**0.03** (**0.04**|0.64)
$${p}_{tc}$$				
Age	0.35(0.12|0.43)	0.10 (**0.01**|0.96)	0.07 (0.17|0.27)	**0.02** (**0.00**|0.58)
HR	0.06 (0.05| 0.46)	0.46 (0.50|0.69)	**0.01** (**0.00**|0.21)	**0.00** (**0.00**|0.16)
CBF	0.31 (0.30|0.70)	**0.01** (**0.04**|0.15)	0.30 (0.51|0.45)	**0.00** (**0.00**|**0.03**)
Velocity	0.39 (0.76|0.33)	0.74 (0.35|0.61)	0.19 (0.18|0.60)	0.41 (0.50|0.49)
Quality	0.40 (0.28|0.68)	0.67 (0.47|0.30)	0.68 (0.82|0.70)	0.09 (0.07|0.85)

In general, combining all root data revealed the most significant trends, where ${p}_{tc}$ was the only measurement that significantly correlated with age, heart rate, and CBF.

#### Global trends

**Figure 4:**
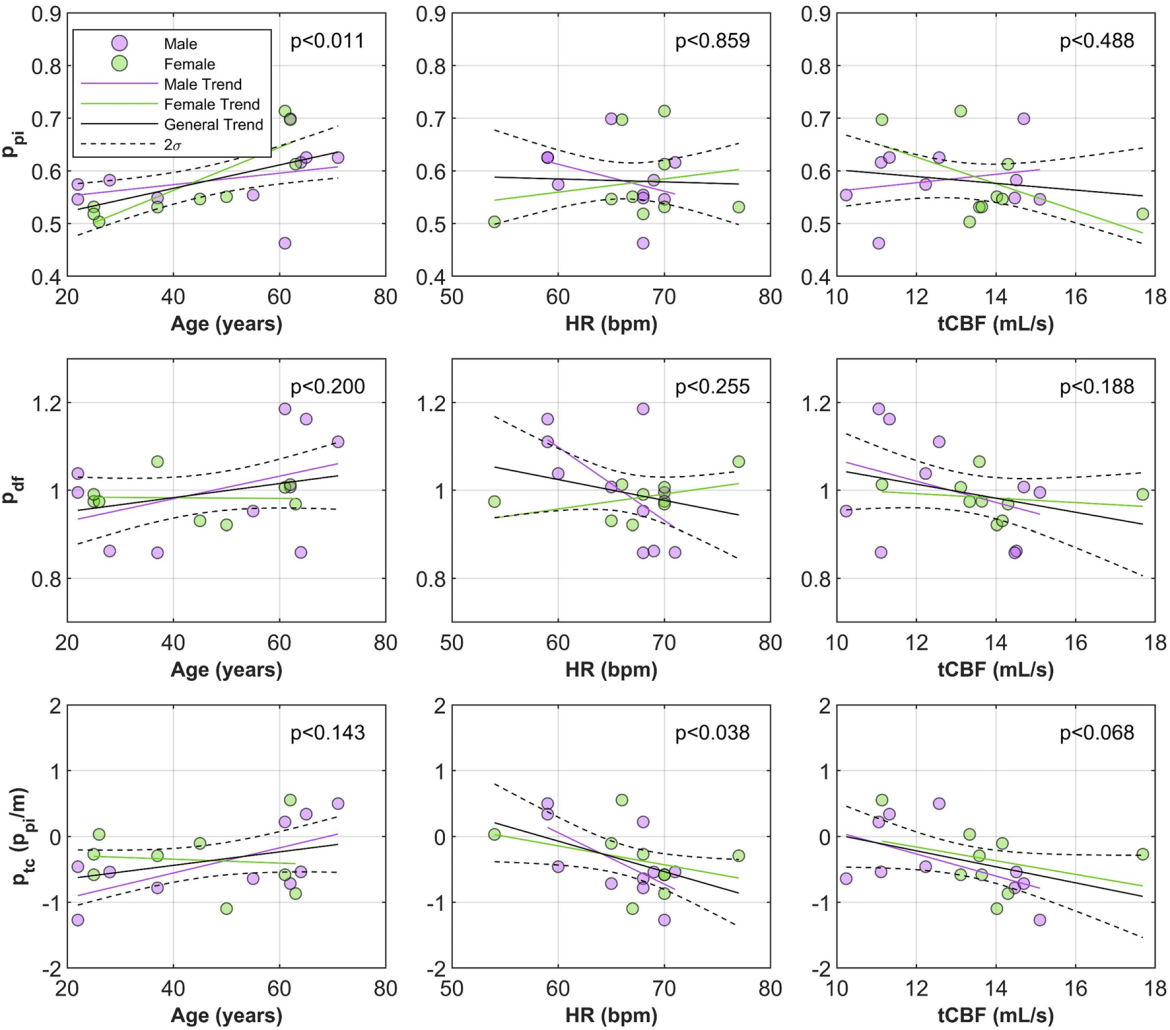
Trends for each PWE index: ${p}_{pi}$ (top row), ${p}_{df}$ (middle row), and ${p}_{tc}$ (bottom row) values with respect to age (left), heart rate (center), and cerebral blood flow (right). These values are the average indices of all three roots. Abbreviations: heart rate (HR), total cerebral blood flow (tCBF).

We now establish whether root trends can be combined as a global cerebrovascular index. Significant trends observed at the root level (age, heart rate, and CBF) flow are now compared when all three root indices are averaged. All three indices increased with age as expected (see Fig. 4 for plots against age, heart rate and CBF). All three indices showed a decreasing trend with increasing heart rate but only significantly for ${p}_{tc}$. To quantify the difference, each index was normalised with a zero mean and division by standard deviation. The trends were then refitted, the smallest dependence on heart rate was ${p}_{pi}$, then ${p}_{df}$ with a slope 1.47 times larger, and the strongest dependence was ${p}_{tc}$ with a slope 2.8 times larger than ${p}_{pi}$. ${p}_{pi}$ and ${p}_{tc}$ identified sex trends for age, where ${p}_{pi}$ differences only appear in later life, contrary to ${p}_{tc}$ sex differences which appeared more in mid-life. Negative male heart rate trends were significant for both ${p}_{df}$ and ${p}_{tc}$. No global index was significantly correlated with CBF although ${p}_{tc}$ was closest to significant. A table summarising the p-values for each global trend is available in Table 3.

**Table 3:** *p* values of all slopes for each global, root averaged index regressed against age, heart rate, and CBF. Significant values at a threshold of *p* < 0.05 are bolded. *p* values first show the general trend, and then bracketed (male|female) trend. Abbreviations: cerebral blood flow (CBF), heart rate (HR).

	${p}_{pi}$	${p}_{df}$	${p}_{tc}$
*p* value of trend			
Age	**0.01** (0.24|**0.00**)	0.20 (0.24|0.94)	0.14 (**0.04**|0.80)
HR	0.85 (0.28|0.61)	0.26 (**0.05**|0.19)	**0.04** (**0.04**|0.35)
CBF	0.49 (0.52|0.12)	0.18 (0.31|0.61)	0.07 (0.11|0.34)

## Discussion

This work aimed to produce a more robust pulsatility based index to study the risk of PWE. The index we propose integrates hundreds of ${p}_{pi}$ values, as opposed to a handful at select locations from previous work on the topic. We analysed root and global trend dependencies with several possible covariates and identified more consistent results and a higher alignment with knowledge on hemodynamic physiology for ${p}_{tc}$, suggesting ${p}_{tc}$ as a positive step towards characterising pulsatility-based hemodynamics. ${p}_{tc}$ theoretically should be less prone to spatial bias than previous indices (as identified in Fig. 2 with pulsatility variability). Furthermore, the measurement is locus independent, enabling a direct comparison between subjects independent of anatomical variation. This enables a cross-subject comparable PWE characterisation using pulsatility data which may translate in robust analyses of participant groups (understanding the differences between age or sex groups for instance) and linking PWE with specific conditions or medical outcomes. This measurement is currently only possible by fully leveraging 4D flow data within an automated processing pipeline, in our case, QVT, which we enhanced during the research process available as QVT + .

### Algorithm and fitting

To measure ${p}_{tc}$, we first developed a module for QVT to process the new index which adds ~ 1 min to the already quick processing time of ~ 7 min per subject using a standard processor. This contrasts with the average processing time of commercial 4D flow packages that takes between 22 and 51 min to manually sample 6 vessel locations^33^ while QVT collects hundreds. After simple initialisation, the module runs an algorithm to reconnect isolated vessel segments. The influence of connectivity errors based on kissing and crossing vessels was minor, adding a < 3% error to the measured slope (${p}_{tc}$). At times, parameter tuning was needed to fully connect the vascular networks. This was acceptable, though the algorithm could be enhanced to have a dynamic search distance at the cost of increasing the amount of initialisation data. Because our connectivity algorithm does not evaluate branch compatibility at a flow level, it was possible that veins were included in our network. During our manual curation of the connectivity, no veins were identified and a final visualisation of our connected arterial network (Fig. 2) was consistent with other networks that specifically pre-processed to exclude veins^11,28,32,34^. It is possible however that with different data or measuring transmission in more distal arteries, that this exclusion may be necessary for the connectivity algorithm to account for, perhaps by identifying large temporal shifts in flow peaks at bifurcations identifying incorrectly connected segments or following existing methods^28^.

To exclude outliers and rely on high-quality data, a quality function $Q(\mathbf{x})$ was proposed. This clearly identified incorrectly segmented cross-sections and noisy velocities that were rightfully excluded from our analysis. The minor percentage excluded still left plenty of data to inform our fit. Certainly, the quality function can be improved by taking into account estimations of partial volume effects based on vessel radius, SNR estimates based on raw velocities, and tuning the individual contribution of each quality metric via a quality-based weight. At this stage, the minimal influence of the weighting showed that the fit was still on the right track to at least exclude the lowest quality data.

When evaluating the fitting procedure (see Fig. 2), important observations were that the linear hypothesis made based on Padmos et al.^31^ was visually acceptable. Interestingly, the ${p}_{pi}$ data were more variable than the data presented in Padmos et al. using computational 1D modelling. Given the ${p}_{pi}$ insensitivity to partial volume effects^28^, the robustness of QVT^30^, and our own calculated acceptable quality, we hypothesise that the ${p}_{pi}$ variability shows physiological vessel variability. 4D flow cannot identify small vasculatures branching from large vessels, so this variability may be caused by more complex flow dynamics that are not accurately captured in the computed cross-sectional 4D flow. Another possibility is that with increasing vessel depth, the blood velocity decreases which would decrease the SNR and potentially over, or underestimate pulsatility. Work by Vikner et al.^28^ showed that low velocity distal waveforms were recoverable with large sample averaging, and we expect that in this work the increase in samples with depth enhanced the recovery of trends.

We observed oscillations in pulsatility along the length of the vessels. In the root vessel of the ICAs, this variability has been identified by van Tuijl et al.^35^ identifying an increasing pulsatility through the carotid canal, and then damping though the cavernous sinus. As for distal oscillations beyond the circle of Willis, initial examination showed that this was not related to the curvature of the vessels and, at times, correlated with $Q$. It is possible that these oscillations are temporal artefacts caused by collection timing missing peak flow dynamics. More investigation is needed, which is beyond the scope of this article. Regardless of origin, these oscillations present a source of error in our linear slope fitting procedure, becoming more relevant as number of cross-sections decrease. Future work involving field of view ablation tests are warranted to see the sensitivity of transmission to coverage, similar to Björnfot et al.^34^ and perhaps even excluding specific vessels with known oscillation patterns.

One issue with a linear approximation is that extrapolation to deeper tissue would maintain that trend, i.e., a positive, or shallow slope would predict extremely large pulsatility in the capillaries. This is likely not the case, and at some stage, due to continued bifurcations, changes in mechanical properties, or strength of myogenic response, the pulsatility would eventually be dampened. It is, however, unclear at what scale that would happen. Studies using computational models, including deep vasculature, may inform us in the future and, with that knowledge, the ${p}_{tf}$ function can always be updated to better capture the variance of the data and any non-linearities that may appear as more cases are processed.

### Interpretation of root and global trends

We now discuss the observed trends of each covariate individually however, we acknowledge that the sample size is small, and that this is only early evidence; larger studies must be done to assess the consistency and proper interpretation of these trends.

#### Age trends

All indices increased with age, strongest in ${p}_{pi}$ and ${p}_{tc}$. This was anticipated as all pulsatility based literature agrees that age is a strong covariate. For ${p}_{pi}$, we identified that females had lower pulsatility in youth which increases faster with age than in men, in agreement with some literature^36^, although other works have shown no sex differences^14,15^. For ${p}_{df}$, literature suggests females dampen less than males, especially in younger subjects^15,36^. Since ${p}_{tc}$ is effectively a large-scale measure of damping (or transmission we term here), it makes sense to expect similar trends, which we observed. Interestingly, our measured ${p}_{df}$ did not agree with this literature. We suspect that this highlights the differences in modality between MRI ${p}_{df}$ and TCD ${p}_{df}$ which these studies used. It is also possible that the small sample size is impacting the ability to identify trends in ${p}_{df}$. We saw numerous damping examples that would make incorrect measurements due to local vessel pulsatility variability, and only at large sample size would that noise average out like the works of^15,36^. This may be highlighting that ${p}_{tc}$ may have improved the measurement specificity, matching expectations in even a low sample number. Conversely, ${p}_{tc}$ may have happened to be statistically significant at this small sample size.

Interpreting the magnitude of the indices with age; unexpectedly, all root ${p}_{pi}$ indices were in a healthy range (${p}_{pi}$ < 1). Although this is a healthy cohort, it is reasonable to assume that some participants who still present as healthy may present in a pathological range similar to Roberts et al.^14^. We identified at-risk participants in ${p}_{df}$ and ${p}_{tc}$ that have elevated indices. For these indices, ${p}_{df}$ predicted 50% of the cohort was elevated (${p}_{df}$ > 1) with many mid-life (20–60) participants included as well. This is unlikely, and by individually analysing participant trends, many ${p}_{df}$ measurements were expectedly spatially biased, i.e., the chosen vessel was an outlier of the global trend. This is observable in Fig. 2 where the ratio of proximal to distal would clearly disagree with our fit. ${p}_{tc}$ identified assumed elevated participants, and most were in late-life (60 +), which is an age range they are more likely to present.

#### Heart rate trends

Regarding heart rate, it has been shown using computational models that faster beating (within resting 40-80bpm) leads to more stable blood flow and lower cerebral ${p}_{pi}$ , whereas slow beating increases ${p}_{pi}$^37^. This was also shown experimentally in patients recovering from transient ischemic attacks or minor stroke^10^. We expected this to manifest for ${p}_{pi}$ directly, and due to increased ${p}_{pi}$ at low heart rate, we expect a decrease in damping and an increase in transmission (${p}_{df}$ > 1, ${p}_{tc}$ > 0). This heart rate dependence was only significantly observed for ${p}_{tc}$, and particularly for males. Females may not present significant trends because their cardiac contractility is higher overall^38^, altering the dependence on heart rate. Interestingly, no pulsatility studies with exception to Webb et al.^10^ have presented relationships with heart rate. Our work also highlighted the locus-dependent nature of pulsatility (Fig. 2). This variability has likely caused significant masking of any location specific pulsatility trends with heart rate, and sex to date, explaining the lack of significant trends in ${p}_{pi}$ and ${p}_{df}$.

#### Cerebral blood flow and velocity trends

We studied the impact of low CBF as a source of pulsatility over or underestimation in our measurement. CBF did cause an impact, with higher transmission and less damping at lower flow magnitude. Lower CBF could mean lower flow and lower pulsatile amplitude, resulting in a lower vascular dampening and, consequently, a less pronounced transmission. This would have likely manifested in our comparisons with pulsatility and CBF, but it was not the case (no statistically significant correlation). An alternative explanation is that, at low CBF, the pulsatility is larger due to either one or more of the following reasons: (i) a slower heart rate with increased cardiac output, (ii) a lower diastolic stressed volume which is increased during systole (i.e., the lower heart rate results in more unloading of the stressed component of the vessels), or (iii) increase in peripheral resistance. More work is needed to properly characterise the relationship between these factors and CBF changes. A better measure would be relative flow, normalised by total perfusing luminal volume, or by proxy, perfused tissue volume. This would be identifiable on a root-specific basis with vessel selective arterial spin labelling for instance^39^. A relative measure of CBF would help understand whether transmission and damping are associated with hyper or hypo perfusion at low flow magnitude. The former would support the observed increase in transmission.

The low flow trend also helps explain the increased transmission in the BA networks which have lower flow than ICAs. However, it may also be due to vascular distance from the heart, differences in vessel wall mechanical properties, or geometrical differences between ICAs and BA. For instance, the rather straight BA versus observed damping in the curved ICAs^20^ may play a role, although since this is a network-based measurement, it is anticipated local regions of damping will not greatly impact the root trend. For the left and right ICA differences, the minimal influence due to CBF and ${p}_{pi}$ suggests that it may measure an asymmetric PWE risk. This may highlight an increased risk of vascular events in the vascular tree with a more positive ${p}_{tc}$, although research is needed.

Importantly, the lack of significant trends with velocity highlight shallow slopes were not influenced by a decreased SNR; female data were potentially even higher quality than males as we observed a larger velocity in females. However, with identical CBF, females potentially have smaller luminal volume if peripheral resistance is constant. Smaller luminal volume would cause an increase in partial volume effects but the initial work of Vikner et al.^28^ suggests pulsatility is not influenced by this effect. More work is needed to understand these sex differences with a first step to include measuring haematocrit to characterise differences in peripheral resistance.

#### Quality trends

Finally, we observed trends for ${p}_{pi}$, ${p}_{df}$ and ${p}_{tc}$ with mean quality. We suspect significance of ${p}_{pi}$ was simply due to chance, however, for damping and transmission, since quality is a measure of data stability, low quality overall highlights the general stability of the data to perform hemodynamic measurement. Higher damping and transmission were associated with lower quality. This would help support the confidence of a particular measurement and quantitatively represent a metric of acquisition quality of 4D flow overall. Perhaps any amplification is non physiological beyond the circle of Willis and strictly due to data quality, however, abrupt changes in geometry or mechanical properties can change pulsatility, highlighting further refinement of quality in the future to help interpret measurements.

#### Global index impressions

As a global index, many features were preserved when averaging all roots. ${p}_{tc}$ maintained nearest significance for all root trends, age, heart rate, CBF, when considering interpretable sex differences, and seems well suited to compare with other global measures of cerebrovascular risk and degeneration in continued studies.

### Alternative ${{\varvec{p}}}_{{\varvec{t}}{\varvec{c}}}$ applications

An interesting application is the use of ${p}_{tc}$ to personalise patient computational models of cerebrovasculature. As Padmos et al.^31^ showed, pulsatility transmission is based on the assumed vascular compliance of the model. These compliances could be adjusted to match ${p}_{tc}$, increasing the patient specificity of the model. Transmission could also be measured in veins, although appropriate changes to the quality function should be made, for instance in valuing circularity, which is not appropriate for collapsible vessels or the triangular cross-section of sinuses for instance. The concept of transmission is applicable to vasculature outside the brain as well, provided distance and pulsatility are available. From a software perspective, the functions developed would work out of box. Lastly, van den Kerkhof et al.^4^ observed that there were local correlations between pulsatility and the number of imageable perivascular spaces. Our global ${p}_{tc}$ index would not capture these subtleties; however, by choosing a new root deeper into the vasculature, a more local ${p}_{tc}$ or even vessel-specific transmission can be measured. The limitation of how many points are needed for a robust slope will need to be explored.

### Perspectives

The difficulty of developing pulsatility-based indices are that they depend on so many hemodynamic factors. We identified several in this work: heart rate, age (proxy for arterial stiffness), sex, flow magnitude, but other factors not included are also expected to alter interpretation (for example, cardiac contractility, terminal resistance, pressure magnitude, mechanical properties, geometry, haematocrit, and active modulators of vascular tone). A quality index should be sensitive to the above, and interestingly, selective vessel pulsatility, the most widely studied, had the least meaningful correlations with what we measured. This is particularly relevant for TCD studies; while selective pulsatility may be relevant for certain research questions, associating pulsatility with global metrics should be treated with caution. While some covariates are not feasibly measurable to fully characterise the pulsatility state, studies that include proper cardiac characterisation, in addition to cerebral, may help distinguish the benefit of cerebral data. As well, physiological perturbations that alter cerebrovascular tone^40^, for instance carbon dioxide, and cyclooxygenase inhibitors are great first steps to characterise the vascular physiology involved in damping^41–43^.

In our opinion, an index that includes total vascular network information (as we present in this work) is the best approach to characterise comparable cerebral hemodynamic properties across-subjects. Tangent work leveraging arterial network 4D flow to measure pulse wave velocity intracranially^32,34,44^ -which is also related with neurodegeneration^8,34,45^- is perhaps a synergic complement to our proposed index to enhance the identifiability of PWE.

To better leverage distal hemodynamics, dual velocity encoding sequences should be explored to maximise low velocity SNR^46^. That would help identify whether linear transmission approximations are still relevant or if new trends manifest. Lastly, we observed multiple excluded cross-sections due to failed automated segmentation. It is impractical and prone to error to manually segment each participant, and while our quality function would identify the critical regions, these may be minimised with advances in segmentation.

## Conclusion

This research presented pulsatility transmission ${p}_{tc}$ as a new CBF pulsatility-based index to study PWE risk. It is measured by characterising vascular pulsatility as a function of distance in the vascular network provided by 4D flow. We compared this measure with pre-existing ${p}_{pi}$, and ${p}_{df}$ indices and concluded that ${p}_{tc}$ captured the most consistent and interpretable dynamics across age, heart rate, sex, and CBF magnitude. Future work with a larger cohort and cohorts with several pathologies is needed to further explore this index. A benefit towards this end is that ${p}_{tc}$ can be retrospectively measured with any 4D flow data. To engage the community with these data, we have built and made the measurement software available on an open-source platform.

### Supplementary Information

Supplementary Information.

## Data Availability

The raw scan data are available upon request to G.D.M.T. and S.J.H., after appropriate institutional data sharing and ethics agreements have been met.
