# Supplementary material for: Measuring global cerebrovascular pulsatility transmission using 4D flow MRI
Source: Sci Rep. 2024 Jun 1;14:12604. doi: 10.1038/s41598-024-63312-4 (PMC11144255; doi:10.1038/s41598-024-63312-4)
Supplement: Supplementary file 1 — Supplementary Information. [file 41598_2024_63312_MOESM1_ESM.pdf]

Scientific Reports

Supplementary: Measuring Global Cerebrovascular Pulsatility Transmission Using 4D Flow MRI

Sergio Dempsey[1,*], Soroush Safaei[1], Samantha J. Holdsworth[2,3], Gonzalo D. Maso Talou[1]
[1]*Auckland Bioengineering Institute, University of Auckland, Auckland, New Zealand*
[2]*Mātai Medical Research Institute, Tairāwhiti Gisborne, New Zealand*
[3]*Faculty of Medical and Health Sciences & Centre for Brain Research, University of Auckland, Auckland, New Zealand*
Correspondence*:
Sergio Dempsey
sdem348@aucklanduni.ac.nz

1 SUPPLEMENTARY NOTE 1:CONNECTIVITY ALGORITHM AND USER INITIALISATION

For each vessel i, numbered $1, \ldots, N$, the cross-sectional planes within that vessel are ordered in the direction of flow from $\mathbf{x}_1^i, \ldots, \mathbf{x}_n^i$, meaning that $\mathbf{x}_1^i$ is vessel i inlet and $\mathbf{x}_n^i$ is the outlet. Using all $\mathbf{x} \in \mathcal{V}$, we designed a breadth-first search algorithm to reconnect vessels for any starting $\mathbf{x}^r$.

Initialise connectivity matrix $C_{R,G}$ with root vessel r where R is row, and G is generation. $C_{1,1} = r$, $Gen = 1$.

while $flag = 1$ **do**

  for each nonempty R in $C_{R,G}$ where $G = Gen$ **do**

    find all $|\mathbf{x}_{[1,n]}^i - \mathbf{x}_n^{C_{R,Gen}}| \leq Dist$

    store nearby terminals as subset $\mathbf{k}$ vessel numbers.

    if all found $\mathbf{x}_{[1,n]}^{\mathbf{k}}$ are inlets **then**

      insert length of $(\mathbf{k} - 1)$ rows at R and insert $\mathbf{k}$ to $C_{R,Gen+1}$

    else

      if $\mathbf{k}$ is nonempty **then**

        outlets are nearby which implies a vessel crossover or kissing.

        for each vessel in $\mathbf{k}$ **do**

compute $|\mathbf{x}_1^k - \mathbf{x}_n^{C_{R,Gen}}|$ as $\dot{\mathbf{x}}^{\mathbf{k}}_{\mathbf{gap}}$ ($\dot{x}$ is tangent derivative)

compute **dot**($\dot{\mathbf{x}}^k_{gap}$, $\dot{\mathbf{x}}_n^{C_{R,Gen}}$)

compute **dot**($\dot{\mathbf{x}}^k_1$, $\dot{\mathbf{x}}_n^{C_{R,Gen}}$)

store mean dot product

end for

find maximum mean dot product vessel (k_{dotmax})

insert $C_{R,Gen+1} = k_{dotmax}$

remove $\mathbf{x}^{C_{R,Gen}}$ and $\mathbf{x}^{k_{dotmax}}$ from $\mathbf{x}^i_{(1,n)}$ so the other outlet will collect all remaining inlets on next pass.

else

No terminals found, do nothing.

end if

end if

end for

if New Connections Made **then**

$Gen = Gen + 1$

else

$flag = 0$

end if

end while

A completed example connectivity matrix C is
$$\begin{bmatrix} 70 & 55 & 30 & 28 & 82 \\ & & & & 36 \\ & & & 87 & 15 \\ & & & & 23 \\ & & 20 & 53 & \\ & & & 11 & \end{bmatrix}$$
where each number represents an isolated vessel ID number, and the interpretation is as follows. For each vessel ID, every space under that number represents a row with vessels connected to that parent vessel ID. Vessel 70 is the root to which all vessels are connected. On the first bifurcation (column 3), the vessels beyond and within rows 1-4 represent the subtree connected to vessel 30, a similar argument for vessel 20.

We have built a module in QVT+ to initiate the connectivity algorithm, which is then run separately (also available with QVT+). The module is an interactive table where vessel branch numbers and positions can be input after selecting appropriate locations on the QVT interactive vasculature. An example is shown

in Figure S1 of this interactive table that opens alongside standard QVT. For the p_{tc} analysis, a selected number of cells must be filled (P1:P4) (refer to Figure S1 for numbering):

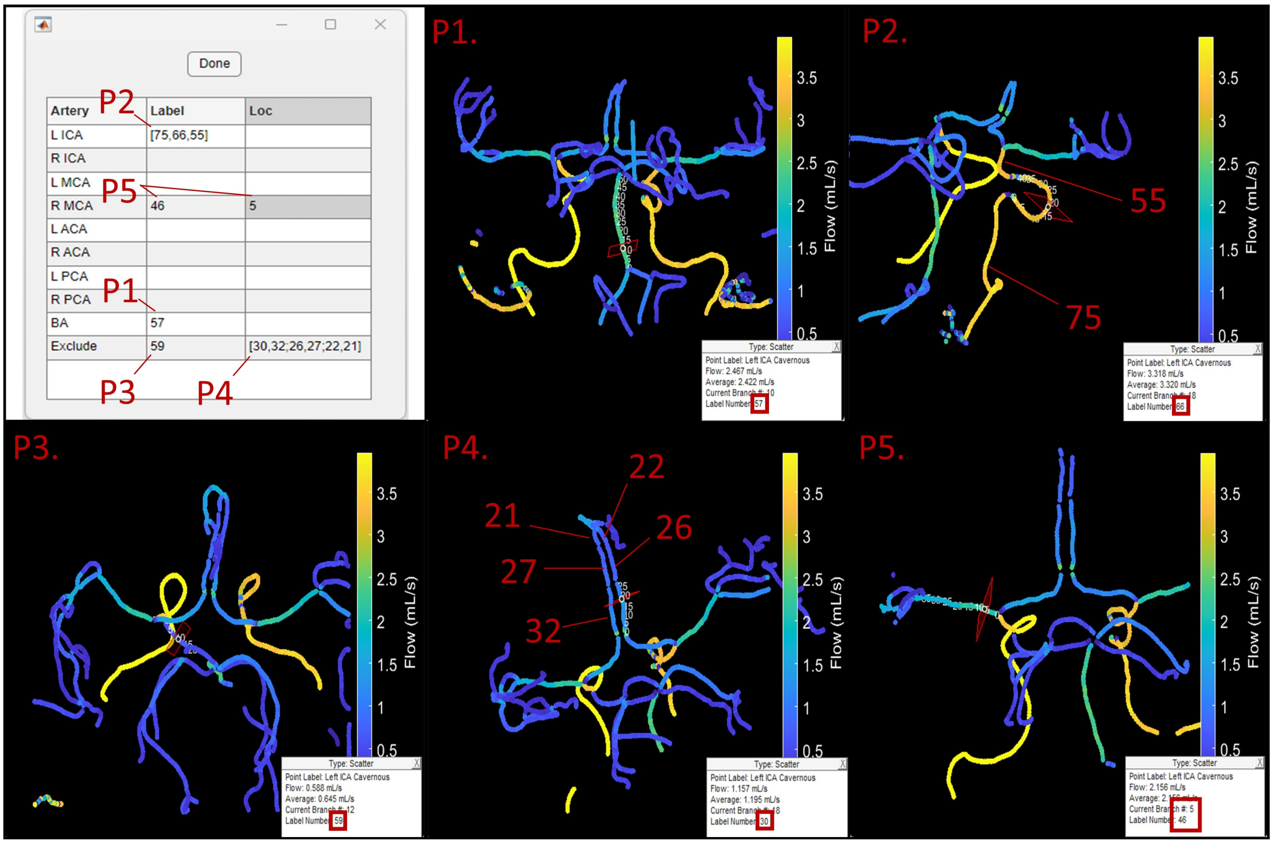

Figure S1: QVT+ table for the algorithm initialisation. Points 1 to 5 show how the table is filled out for the estimation of p_{tc}.

- **P1:** Each root vessel value must be input (ICAs and BA). Typically, only one value is needed in each root.
- **P2:** If, for instance, the carotid siphon folds on itself and causes non-physiological branches in QVT, a connected vessel number array can be entered.
- **P3:** In the "Exclude" row, communicating arteries must be included; otherwise, the algorithm will connect vessels from the ICA to the PCAs. Other vessels can be inputted (as an array format) to stop the connectivity algorithm if desired.
- **P4:** In the case where the ACAs are close together, incorrect hemispheric crossovers can occur in the algorithm. To avoid this, the left and right vessel numbers can be supplied for the ACAs, respectively.
- **P5:** Optionally, for selected CoW vessels, the vessel number and the centreline number can be entered; this can be used to output local p_{pi} for other analyses, but it is not necessary to initialise the p_{tf} algorithm.

2 SUPPLEMENTARY NOTE 2: SHIFTED VESSEL SIMULATION

This section describes the simulation used to evaluate errors in $p_{\mathbf{tc}}$ caused by uncertainty in distance ($d(\mathbf{x}^r, \mathbf{x})$) estimates. Specifically, the distance uncertainty is due to curvature simplifications or incorrect vessel connectivity at kissing and crossing junctions. The occurrence of these errors manifests itself as random shifts in the distance $d(\mathbf{x}^r, \mathbf{x})$ for the points of the daughter vessel and compound as more crossovers are encountered.

To simulate this error, synthetic vessel segments were generated along the distance d. A vessel segment for this purpose is a random length of a vessel with several $p_{\mathrm{pi}}(\mathbf{x})$ measurements evenly sampled along the length of the segment, as encountered in QVT. In this synthetic case, the $p_{\mathrm{pi}}(\mathbf{x})$ trend within the vessel follows the exact $p_{\mathrm{tf}}(d)$ function, which for this case is $p_{\mathrm{pi}}(\mathbf{x}) = -1 \times d$. The algorithm is designed to increase the density of segments and then eventually decrease to match the typical point density in 4D flow vascular networks (Björnfot et al., 2021). Once a vessel segment has been simulated, a random shift (error) in position is assigned that also increases with distance. This is added to match the expected compounding shift errors. An example plot of the synthetically generated data is shown in Figure S2. This point cloud is then fitted with a first-degree polynomial, similar to our $\mathrm{p_{tf}}$ function using a least-squares method in Matlab (R2023a). For 1000 simulations, a mean slope of $-0.99998 \pm 0.027\%$ quantified the shift error. The code to run this simulation is available on the QVT+ GitHub.

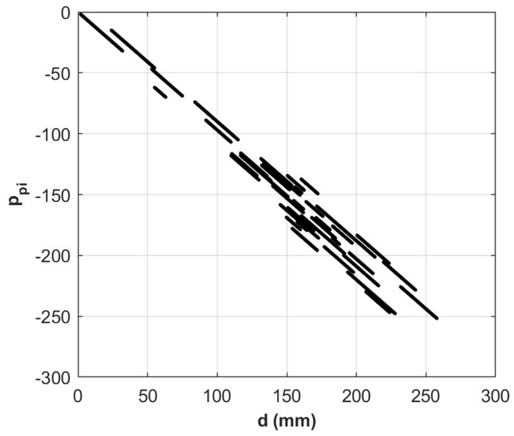

Figure S2: Synthetically generated data with local -1 slope. The data segments representing the pulsatility in a given branch are shifted to match the expected sources of error in the vessel connectivity algorithm.

3 ADDITIONAL FIGURES AND TABLES

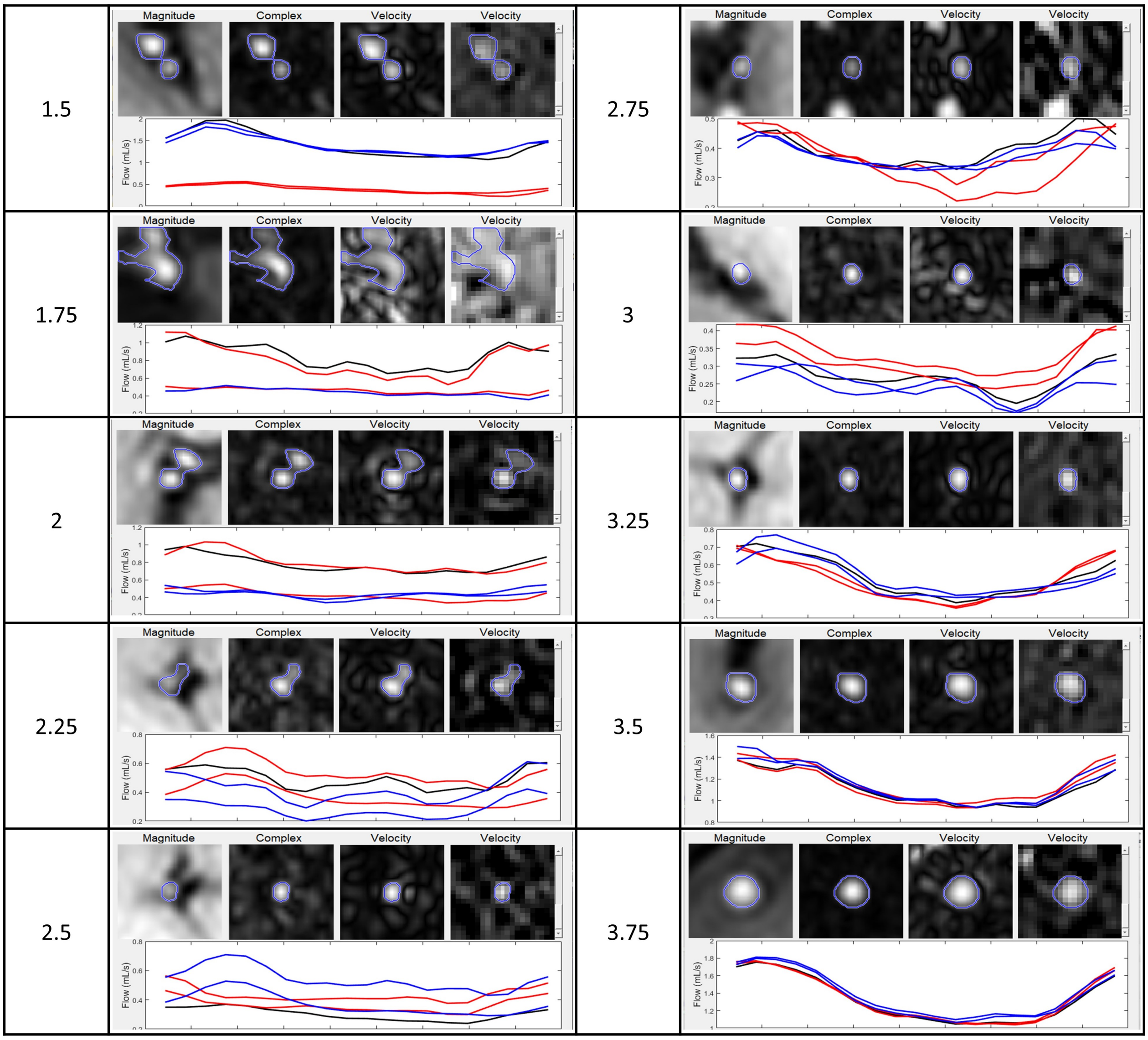

Figure S3: Examples of segmentation and local flow traces at several quality levels ranging from 1.5 to 3.75.

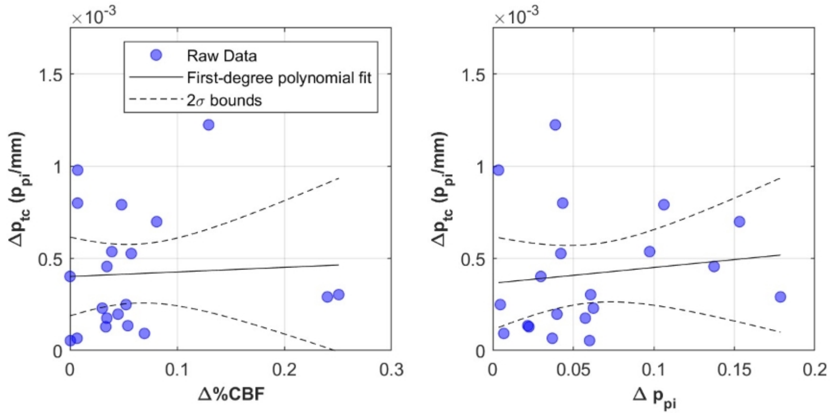

Figure S4: Plots of the left and right ICA difference in p_{tc} with respect to the difference of left and right % cerebral blood flow (CBF) (left), and p_{pi} difference (right).

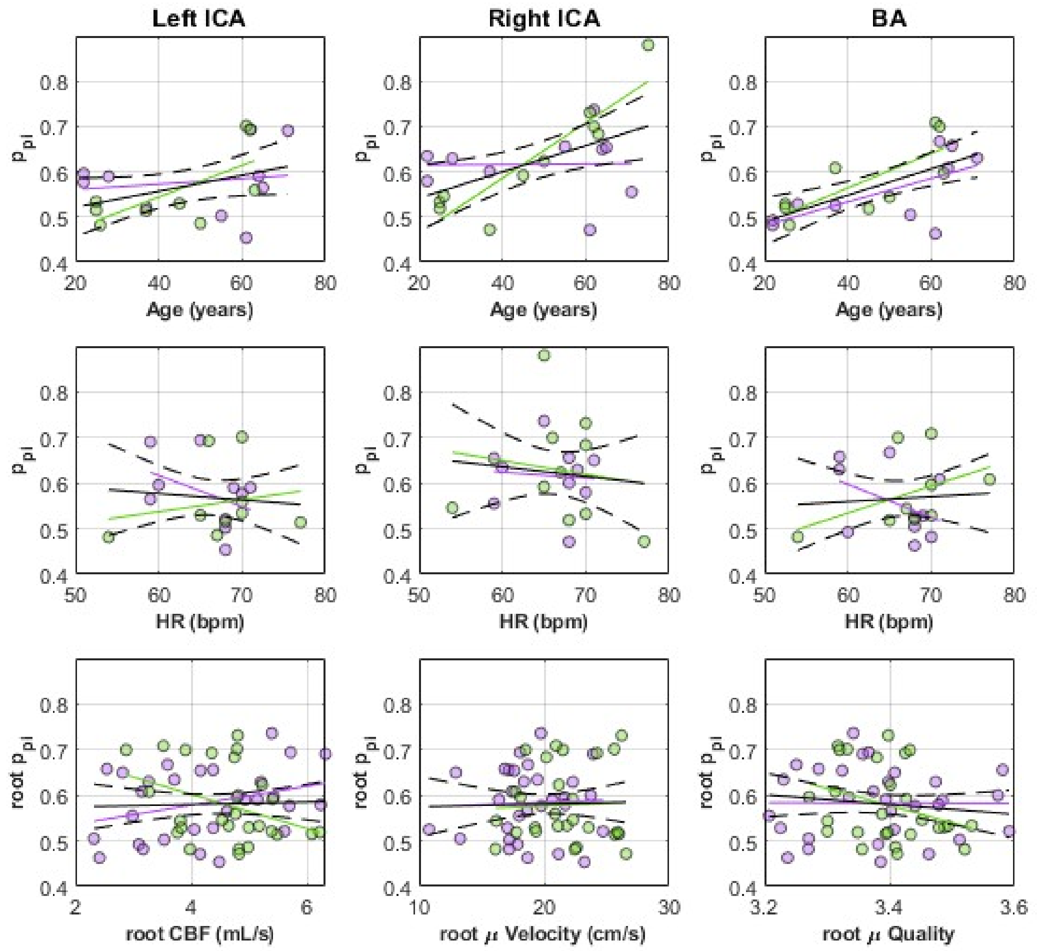

Figure S5: Individual root trends of p_{pi} with respect to age (row one), heart rate (row two) for the left ICA (left), right ICA (centre), and BA (right). All roots are then merged for other comparisons against mean CBF, velocity, and quality (bottom row).

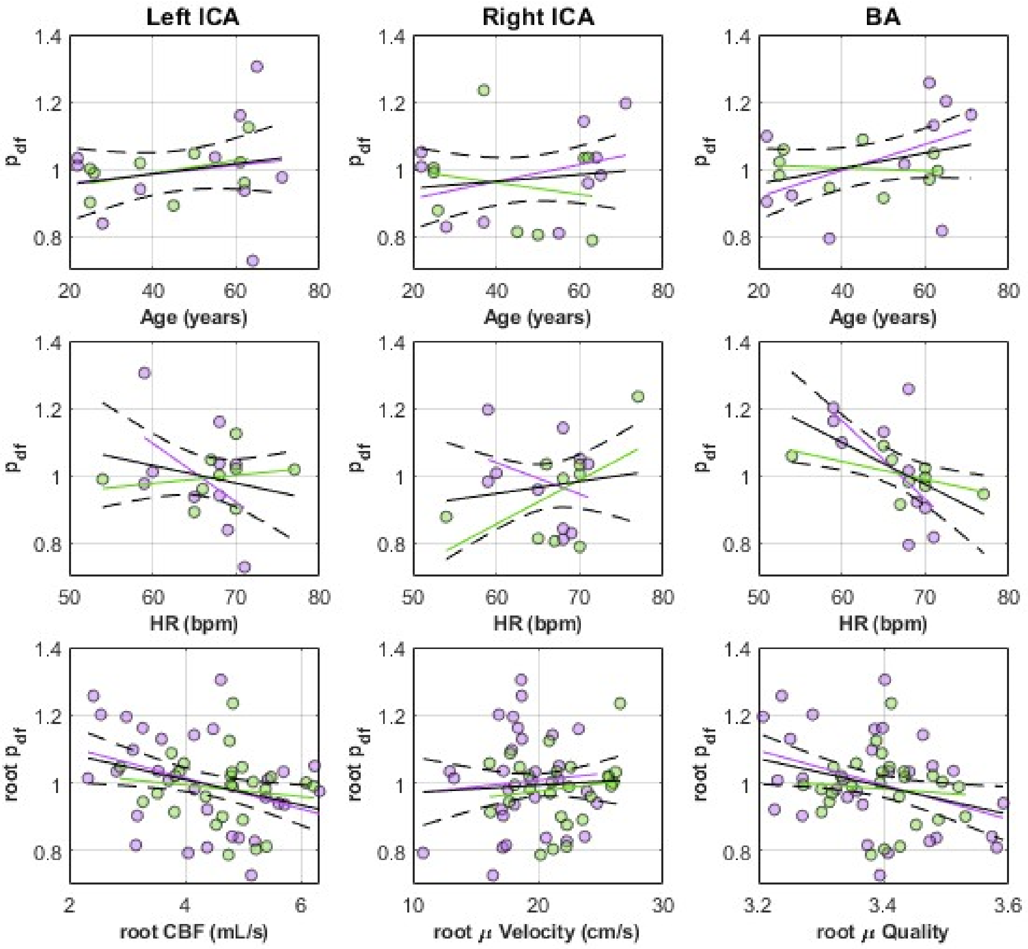

Figure S6: Individual root trends of p_{df} with respect to age (row one), heart rate (row two) for the left ICA (left), right ICA (centre), and BA (right). All roots are then merged for other comparisons against mean CBF, velocity, and quality (bottom row).
